# Naturally Occurring Triploidy in Cannabis

**DOI:** 10.3390/plants12233927

**Published:** 2023-11-22

**Authors:** Richard Philbrook, Marzieh Jafari, Sydney Gerstenberg, Krista L. Say, Jeremy Warren, Andrew Maxwell Phineas Jones

**Affiliations:** 1 Dark Heart Nursery, 630 Pena Dr. Suite 600, Davis, CA 95616, USA; rphilbrook@darkheartnursery.com (R.P.); sgerstenberg@darkheartnursery.com (S.G.); ksay@darkheartnursery.com (K.L.S.); 2 Department of Plant Agriculture, University of Guelph, Guelph, ON N1G 2W1, Canada; mjafary@uoguelph.ca

**Keywords:** drug-type, flowcytometry, polyploidization, triploid

## Abstract

Polyploidy is a significant evolutionary process in plants that involves the duplication of genomic content and has been recognized as a key mechanism driving plant diversification and adaptation. In natural populations, polyploids frequently arise from unreduced gametes, which subsequently fuse with reduced or unreduced gametes, resulting in triploid or tetraploid offspring, respectively. *Cannabis sativa* L. is a diploid species, but recent work using artificially induced polyploidy has demonstrated its potential advantages in an agricultural setting. Further, recent work has identified that some elite clonal cultivars, vis. Mac1, are triploid, with no indication that they were artificially produced. The current study was conducted to determine if polyploidy is a naturally occurring phenomenon in cannabis and to estimate the frequency of this phenomenon across populations. To do this, the presence of natural triploid individuals was evaluated in 13 seedling populations of cannabis using a flow cytometry analysis. Among the examined populations, natural triploids were identified in 10 groups with an average frequency of approximately 0.5%. The highest frequency of natural triploids was observed in a self-pollinated population at 2.3%. This research demonstrates that polyploidy is a naturally occurring event in cannabis and triploids are present at an average of approximately 0.5%, or 1 in 200 plants. These data shed light on the natural variation in ploidy within cannabis populations and contribute valuable insights to the understanding of cannabis genetics and breeding practices.

## 1. Introduction

Cannabis (*Cannabis sativa* L.) is a multi-purpose plant in the Cannabaceae family that has garnered significant scientific attention due to its complex chemical profile and diverse industrial [1], medicinal [2], horticultural [3], and recreational [4] applications. The plant is composed of various chemotypes with distinct phytochemical compositions, primarily driven by the presence and ratios of cannabinoids, terpenoids, and flavonoids [5]. Despite the growing interest in cannabis cultivation and its expanding legal status in various regions, the scientific understanding of cannabis genetics and breeding techniques lags behind that of many other crops [6]. 

The recent wave of cannabis legalization in various regions has spurred considerable scientific interest in optimizing cultivation techniques, developing new cultivars, and exploring potential medical applications [7]. However, plant breeding technologies in cannabis are still in their nascent stages, with limited information and research accessible to producers in the field [8]. This knowledge gap presents a significant challenge for producers seeking to develop new cultivars with specific traits, such as a higher cannabinoid content, disease resistance, or tailored aroma profiles [6]. A promising approach for cannabis breeding involves the process of polyploidization, in which the number of copies of each chromosome is increased [9,10,11]. In some studies, artificially induced polyploids, and in particular triploids, have been reported to have an increased yield and cannabinoid content [12]. However, other reports indicate that polyploidy has variable impacts on plant growth and development, likely due to the genetic variation in the starting material, and much more work is needed [13]. 

While cannabis is typically a diploid species, instances of presumably natural triploid (3×) [14] and tetraploid (4×) [15] plants have been observed in some studies. Further, some elite clonal cultivars used in commercial production have been identified as triploids (i.e., Mac1), with no indication that they were artificially produced (unpublished data). Despite their rarity, naturally occurring polyploid cannabis plants offer valuable insights into the plant’s genetic diversity and adaptation to varying environmental conditions [14]. Understanding the genetic consequences of natural polyploidy in cannabis is of significant interest to both researchers and breeders [9]. These polyploid individuals may exhibit distinct traits, such as altered morphology or growth patterns, which could have implications for their ecological niche or potential utility in cultivation [16]. Furthermore, investigating the stability and reproductive viability of naturally occurring polyploid cannabis plants is essential for comprehending the long-term evolutionary dynamics of this species [14]. Research in this area can provide valuable insights into the adaptive potential of cannabis populations and inform breeding efforts aimed at improving various aspects of cannabis cultivation, including the yield, cannabinoid content, and environmental resilience [17].

**Figure 1:**
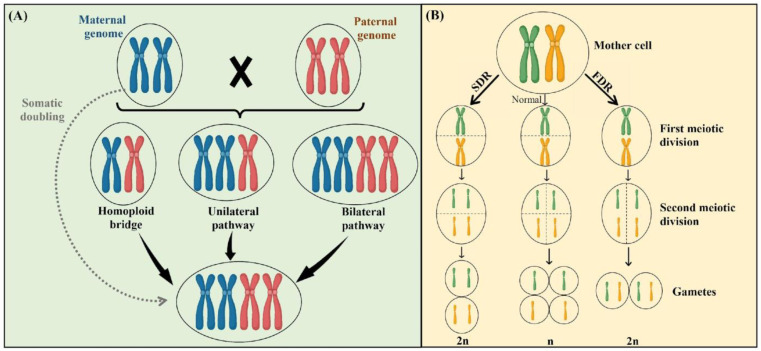
Two main mechanisms lead to the natural occurrence of polyploidy. (**A**) Somatic doubling involves the doubling of chromosome sets within somatic cells, resulting in the development of fully polyploid individuals. In contrast, unilateral polyploidization primarily arises from the fusion of unreduced gametes with reduced gametes, leading to a partially polyploid individual with heterozygous and homozygous regions. Bilateral polyploidization, on the other hand, occurs when two unreduced gametes fuse, generating a fully polyploid individual with complete homozygosity; (**B**) two distinct processes can give rise to the production of unreduced gametes. The first division restitution (FDR) is a meiotic process characterized by the failure of homologous chromosomes to segregate correctly during meiosis I, leading to the production of unreduced gametes with two non-sister chromatids. The second division restitution (SDR) is a meiotic process characterized by the correct segregation of homologous chromosomes during meiosis I but the failure of sister chromatids to segregate appropriately during meiosis II. This figure was prepared using www.biorender.com.

Somatic doubling or the generation of unreduced gametes are the main mechanisms known to result in natural polyploidy [18] (Figure 1). Somatic doubling is linked to mitotic processes (e.g., endoreduplication or endomitosis), which can take place within apical meristematic tissues or zygote cells, ultimately leading to the development of entirely polyploid organisms or mixoploid individuals [19]. However, it is widely acknowledged that somatic doubling is of relatively minor significance in the natural formation of polyploids, despite this being the primary mechanism used for the induction of artificial polyploids [20].

The predominant mechanism leading to polyploidy in plants is understood to involve the production and subsequent fusion of unreduced gametes [20]. In this instance, the unreduced gamete is diploid, containing two copies of each gene, instead of the standard haploid state with only one copy. The ability to generate unreduced reproductive cells is a hereditary trait observed in numerous plant species [19]. In addition to genetic regulation, environmental factors (e.g., nutrient deficiencies, water scarcity, injury, herbivory, and temperature) can exert an influence on unreduced gamete formation [20]. Once created, the unreduced gamete possesses the capacity to merge with another unreduced gamete to produce a tetraploid, a process known as bilateral polyploidization, or with a gamete that has undergone reduction to produce a triploid, termed unilateral polyploidization (Figure 1A) [18]. 

From a cytological perspective, depending on the meiotic stage at which the restitution event occurs, two distinct processes can give rise to the production of unreduced reproductive cells (i.e., the first division restitution (FDR) and the second division restitution (SDR)) (Figure 1) [18]. The FDR encompasses errors that occur during meiosis I. These errors can stem from the failure of homologous chromosomes to segregate correctly during anaphase I and/or the lack of chromosome pairing during the zygotene/pachytene stage (Figure 1B) [21]. Within the SDR process, chromosome pairing and division proceed typically during meiosis I, but an anomaly arises in anaphase II where sister chromatids fail to segregate as expected [22] (Figure 1B). These irregularities facilitate the restoration of the somatic chromosome number within the gametes, leading to the formation of dyads or triads [18]. The genetic outcomes related to the maintenance of heterozygosity vary between each meiotic restitution mechanism [18]. Unreduced gametes produced via the FDR typically contain two non-sister chromatids and may maintain a level of heterozygosity similar to that of their parent cells [21]. In contrast, unreduced gametes resulting from SDR contain two sister chromatids, leading to a lower heterozygosity level compared to their parent cells [22]. The disparity in heterozygosity levels plays a pivotal role in influencing the potential success or failure of a recently formed polyploid, whether within wild populations or in the context of plant breeding [18].

The focus of this study was to determine the extent of natural polyploidy in cannabis. To achieve this, natural diploid crosses were made to generate a series of populations that were screened to determine if they contained any polyploid individuals and estimate the natural rate of occurrence. Naturally occurring triploids, but no tetraploids, were identified at a low rate in most populations. These results clearly demonstrate that polyploidy is a naturally occurring phenomenon in cannabis, likely as a result of unreduced gametes. These results have significant implications for future breeding efforts and help to explain the presence of polyploids within landrace populations and commercial production. 

## 2. Results

**Figure 2:**
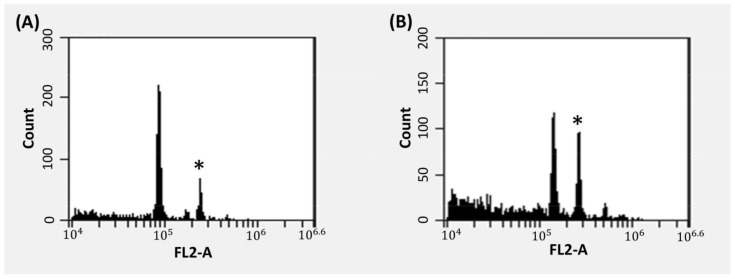
Flow cytometric histograms showing *Cannabis sativa* L. with (**A**) a diploid profile and (**B**) a triploid profile. Peaks indicated with (*) are internal 6n control.

In this study, 13 seedling populations were assessed to identify the occurrence and estimate the frequency of natural triploidy in cannabis using flow cytometry. The FL2-A fluorescence measurement of the total DNA for triploids was recorded at 149,389 (Figure 2B), representing around a 40% increase in the DNA content compared to diploids with FL2-A fluorescence measuring 110,233 (Figure 2A). Among the tested genotypes, the highest observed frequency of natural triploids was in the DHN1 S1 population (Table 1). Subsequently, notable instances of triploidy were observed in the DHN2 × DHN3, Bigfoot Glue, DHN2 × DHN4, DHN5 × DHN3, DHN6 × DHN3, DHN7 × DHN1, DHN8 × DHN9, DHN8 × DHN1, and DHN7 × DHN1 populations. No instances of triploidy were detected in the Jelly Rancher, Gazzurple, and DHN10 × DHN1 genotypes (Table 1).

## 3. Discussion

Polyploidy, an observed phenomenon in the context of plant evolution and diversification, denotes the presence of greater than two complete sets of chromosomes within a single cell nucleus [10]. The assessment of polyploidy prevalence in the scientific literature exhibits substantial variability, with reported frequencies for angiosperms ranging from as low as 30–35% to as high as 70% [18]. Otto and Whitton [23] devised a straightforward method for gauging polyploidy occurrences by examining shifts between even and odd basic chromosome numbers. Employing this methodology, they proposed that polyploidization takes place in roughly 7% of speciation events among ferns and 2–4% in angiosperms. Based on these findings, Otto and Whitton [23] asserted that polyploidy is common and highly probable to represent one of the primary biological events driving sympatric speciation in plants.

While several instances of artificial polyploidization have been documented in cannabis [9,24,25,26], the occurrence of natural polyploidization in cannabis has been reported in only two studies to date looking at landrace populations [14,15]. However, unpublished data has also identified several elite, clone-only cultivars used for commercial production as triploids, without any indication that they were produced through artificial means (unpublished data). Together, these reports suggest that polyploidy is likely a naturally occurring, albeit rare, phenomenon in cannabis. In this study, 10 out of 13 populations contained triploid individuals, while the other three were exclusively diploid. However, it should be noted that the average rate of natural triploidy was around 0.5%, such that the absence of triploids in these three populations was likely a result of the population size and randomness rather than any biological factor. Presumably, if larger population sizes were used, triploids would likely have been identified in all 13 populations. Likewise, while no naturally occurring tetraploids were identified in this study, they have been reported in a landrace population, suggesting that they naturally occur [15], and the absence reported here is likely a product of population size. Based on the data presented here, and assuming male and female gametes fail to reduce at a similar rate, we would expect natural tetraploids to appear at a rate of approximately 0.0025%, or 1 in 40,000. 

The occurrence of polyploids in natural settings enhances heterosis, offering potential advantages for modern breeders [20]. An important merit of polyploidy is its ability to generate seedless triploids [24]. Triploids play a pivotal role in the dynamics of polyploid populations [12]. The incorporation of certain polyploid characteristics could prove advantageous in the cultivation of cannabis [16]. The primary revenue-generating product derived from cannabis cultivation is the unfertilized flower, as seeded plants yield lower quantities and reduced cannabinoid content, rendering them unsuitable for marketing as dried flowers [2]. Cannabis plants are predominantly wind-pollinated, and their pollen can travel considerable distances, resulting in inadvertent pollination issues frequently encountered in outdoor production settings [27]. Even within controlled indoor cultivation systems, concerns persist due to the potential for hermaphroditic plants that can produce viable male flowers, leading to substantial losses due to undesired pollination events [28]. Furthermore, a previous study on artificial polyploidization showed that triploid hemp varieties yielded approximately 30% more than their diploid or tetraploid counterparts, suggesting that triploidy offers advantages beyond seedlessness [24]. However, subsequent research has shown that the impact of polyploidy on cannabis growth and development is variable and likely dependent on the genetic background and combination [13]. Further, we have observed that triploid cannabis plants produced seeds to varying degrees. 

Theoretically, triploids are rendered sterile due to the unbalanced segregation of chromosomes during meiosis, consequently leading to aneuploid gamete production [29,30]. However, in practical scenarios, it is noteworthy that in some species, a significant number of triploids demonstrate the capacity to generate functional euploid gametes, particularly in varying proportions of x and 2x gametes [30,31]. These gametes can serve as valuable contributors, either as female or male parents, in cross-breeding initiatives [32]. For example, in a study on *Ranunculus kuepferi*, Schinkel et al. [33] successfully obtained a total of 125 fully developed seeds from 25 triploid plants. In the context of meiosis in sexual polyploids, the allocation of two chromosome sets from the 2n gamete to a daughter cell tends to give rise to a 2x gamete [30,31]. The occurrence of this chromosome behavior during meiosis is primarily contingent upon the genetic compatibility between the parental contributors of the chromosome sets [32]. In instances where the genetic relatedness is highly close, as observed in sexual autopolyploids, the main configuration of chromosome pairing at diakinesis consists of either trivalents (in the case of sexual autotriploids) or quadrivalents (in the case of sexual autotetraploids) [30,34]. Conversely, when the genetic relationship is further apart, as seen in sexual allopolyploids, the primary configuration of chromosome pairing comprises a univalent and a bivalent (in sexual allotriploids) or two bivalents (in sexual allotetraploids) [31,34]. Lavia et al. [35] observed that the primary chromosomal pairing arrangement in the sexual autotriploid *Arachis pintoi* predominantly featured trivalents, resulting in a pollen grain viability of 42.47%. Ramanna et al. [36] observed that in F1 hybrids derived from Chilean-Brazilian *Alstroemeria* species, there was a simultaneous production of 2n female and male gametes. When these F1 hybrids were self-pollinated, all resulting F2 progeny plants exhibited typical allotetraploid characteristics. Notably, during the metaphase I stages of meiosis, the majority displayed the formation of 16 bivalents, while a smaller proportion exhibited the presence of multivalents [36]. Kovalsky et al. [37] found that triploids resulting from the fusion of 2n × n gametes within the same taxon exhibited more regular meiotic behavior and greater fertility in comparison to triploids with hybrid origins. In the case of Dactylis polyploids, it was observed that natural polyploids demonstrated proficient chromosome pairing during meiosis, whereas their artificial counterparts did not [37]. This observation implies a selective pressure for sexual fertility, potentially serving to stabilize the meiotic process in natural polyploids [30].

## 4. Materials and Methods

### 4.1. Plant Growth Conditions 

High THC cultivars of cannabis were grown in a greenhouse in Half Moon Bay, CA, USA, where they received a combination of natural sunlight supplemented with 400W high-pressure sodium (HPS) lights (Osram, Munich, Germany), delivering a light intensity of 150 ± 50 µmol/m^2^/s photosynthetically active radiation (PAR) at the canopy level. To induce the flowering process, light deprivation tarps were employed, blocking sunlight for 12 h each night for 8 weeks. A specific group of plants was chosen as pollen donors and relocated to shipping containers, where they received 12 h of daily HPS lighting. On the first day of short days, diploid pollen donor plants were sprayed with silver thiosulfate solution at a concentration of 3 mM prepared according to Lubell and Brand [38] to induce male flower development. These plants were sprayed with silver thiosulfate (200 mL/plant) on three occasions, 7 days apart. This treatment stimulated the development of male flowers. 

Female flowering plants, intended for pollination, were also identified and moved to the same shipping containers as the male plants, where they received 12 h of HPS lighting. Once the male flowers were fully developed, the female plants remained in the same containers for two weeks to facilitate controlled pollination. After this pollination period, the female flowering plants were returned to the mixed-light greenhouse with light deprivation tarps for the remainder of the flowering phase. The seeds were considered fully ripened after 8–10 weeks, depending on the specific cultivar.

When the plants reached maturity, they were carefully harvested, and the seeds were collected. These harvested plants were dried under controlled conditions. A custom-designed seed sorter was utilized to collect the seeds for subsequent analysis.

### 4.2. Seed Germination 

*Cannabis sativa* seeds were initiated for germination in a solution of 1% hydrogen peroxide diluted in reverse osmosis (RO) water within 50 mL centrifuge tubes. The tubes were subsequently positioned on a gently oscillating platform at a controlled temperature of 30 °C overnight. Following the emergence of radicles, the seeds were transplanted into 200-cell seed trays filled with a coconut coir potting mix (specifically, Bush Doctor Coco Loco from Fox Farm Soil in Westminster, CA, USA). Subsequently, the seeds were irrigated with sterile RO water, enclosed within a humidity dome, and positioned on a 30 °C heating mat under a continuous 18 h light regimen.

### 4.3. Flow Cytometry Analysis 

To evaluate the total nuclear DNA content, flow cytometry was performed using a BD Accuri C6 flow cytometer (BD Biosciences, Franklin Lakes, NJ, USA). Fresh leaves were collected from healthy plants and preserved in a moist paper towel on ice for a maximum of 24 h before assessment. All materials and specimens were maintained at low temperatures during preparation. About 20 mg of fresh leaves were finely minced on a Petri dish using a razor blade and immersed in 1 mL of Galbraith’s buffer (45 mM MgCl_2_, 30 mM sodium citrate, 20 mM MOPS, 0.1% (*v*/*v*) Triton X-100, pH 7.0 [39]). The suspension of released nuclei was then passed through a 37.4 μm nylon filter into a 2 mL centrifuge tube. The filtrate was stained with 25 µL of propidium iodide stock (1.0 mg mL^−1^) and incubated for 30 min in the absence of light. The duration between sample preparation and flow cytometric analysis (FCM) was about 5 min. The stained nuclei were analyzed with set parameters of 465 V, and data for a minimum of 1000 nuclei per sample were captured within a maximum of 120 s. Relative DNA content was determined utilizing the fluorescence peak area (585/42 nm detector), and fluorescence peak means, coefficients of variation, and nuclei counts were obtained using the BD Accuri™ C6 Software 1.0.264.21. To identify triploid plants, plant tissues with a confirmed diploid status were employed as a standard. To control for pigments and age of tissue causing a slight variation between samples, an internal 6n control was used to localize unknown samples. 

## 5. Conclusions

In this study, we delved into the intriguing phenomenon of natural triploidy in *Cannabis sativa*, demonstrated within a diverse set of 13 cannabis genotypes. Our ploidy determination analysis revealed a clear contrast in the DNA content between triploid and diploid individuals, with a 40% increase in the FL2-A fluorescence measurement, affirming the presence of triploidy in the cannabis genome. Crucially, our investigation identified natural triploids in a substantial majority of the examined genotypes, particularly in the DHN1 selfing population, but this could be a consequence of random variation. These findings underscore the prevalence of natural triploidy in cannabis populations and emphasize its relevance in the field of cannabis genetics. It is apparent that future investigations should delve deeper into the mechanisms underpinning triploidy in cannabis and its potential implications for cannabis breeding and cultivation practices. Of particular importance is the need to explore triploid cannabis sterility and seed development, shedding light on the reproductive biology of the species and helping to develop truly seedless individuals. In sum, the discovery of natural triploidy in cannabis opens up exciting avenues for further research, offering insights that may revolutionize the cannabis industry and its scientific understanding.

## Figures and Tables

**Table 1:** Evaluating natural triploids in 13 different *Cannabis sativa* genotypes.

Cultivar/Cross	Source	Plants Tested	Number of Triploids	Triploid Frequency (%)
Bigfoot Glue	Humboldt Seed Co., Eureka, CA, USA	180	1	0.56
Jelly Rancher	Humboldt Seed Co., Eureka, CA, USA	190	0	0.00
DHN7 × DHN1	Dark Heart, Davis, CA, USA	1449	7	0.48
Gazzurple	Humboldt Seed Co., Eureka, CA, USA	80	0	0.00
DHN2 × DHN3	Dark Heart, Davis, CA, USA	263	2	0.76
DHN2 × DHN4	Dark Heart, Davis, CA, USA	200	1	0.50
DHN5 × DHN3	Dark Heart, Davis, CA, USA	200	1	0.50
DHN6 × DHN3	Dark Heart, Davis, CA, USA	200	1	0.50
DHN8 × DHN9	Dark Heart, Davis, CA, USA	1000	3	0.30
DHN1 × DHN1	Dark Heart, Davis, CA, USA	128	3	2.34
DHN7 × DHN1	Dark Heart, Davis, CA, USA	768	2	0.26
DHN8 × DHN1	Dark Heart, Davis, CA, USA	337	1	0.30
DHN10 × DHN1	Dark Heart, Davis, CA, USA	192	0	0.00
Total		5187	22	0.50 ± 0.603 *

* This value represents mean ± standard deviation.
